# Genomics of neonatal sepsis: has-miR-150 targeting BCL11B functions in disease progression

**DOI:** 10.1186/s13052-018-0575-9

**Published:** 2018-11-29

**Authors:** Li Huang, Lixing Qiao, Huan Zhu, Li Jiang, Liping Yin

**Affiliations:** Pediatric Department, Zhongda Hospital of Southeast University, 210009 Nanjing, People’s Republic of China

**Keywords:** Neonatal sepsis, Differentially expressed genes, Enrichment analysis, Protein-protein interaction network, Regulatory network

## Abstract

**Background:**

Neonatal sepsis is an inflammatory systemic syndrome, which is a major cause of morbidity and mortality in premature infants. We analyzed the expression profile data of E-MTAB-4785 to reveal the pathogenesis of the disease.

**Methods:**

The expression profile dataset E-MTAB-4785, which contained 17 sepsis samples and 19 normal samples, was obtained from the ArrayExpress database. The differentially expressed genes (DEGs) were analyzed by the Bayesian testing method in limma package. Based on the DAVID online tool, enrichment analysis was conducted for the DEGs. Using STRING database and Cytoscape software, protein-protein interaction (PPI) network and module analyses were performed. Besides, transcription factor (TF)-DEG regulatory network was also constructed by Cytoscape software. Additionally, miRNA-DEG pairs were searched using miR2Disease and miRWalk 2.0 databases, followed by miRNA-DEG regulatory network was visualized by Cytoscape software.

**Results:**

A total of 275 DEGs were identified from the sepsis samples in comparison to normal samples. TSPO, MAPK14, and ZAP70 were the hub nodes in the PPI network. Pathway enrichment analysis indicated that *CEBPB* and *MAPK14* were enriched in TNF signaling pathway. Moreover, *CEBPB* and *has-miR-150* might function in neonatal sepsis separately through targeting *MAPK14* and *BCL11B* in the regulatory networks. These genes and miRNA might be novel targets for the clinical treatment of neonatal sepsis.

**Conclusion:**

*TSPO*, *ZAP70*, *CEBPB* targeting *MAPK14*, *has-miR-150* targeting *BCL11B* might affect the pathogenesis of neonatal sepsis. However, their roles in neonatal sepsis still needed to be confirmed by further experimental researches.

## Background

As a kind of inflammatory systemic syndrome that is determined by a serious infection (bacterial or viral sepsis), neonatal sepsis is composed of early-onset sepsis (EOS) and late-onset sepsis (LOS) [1]. The leading risk factors of the disease are prematurity, prolonged rupture of membranes, and low birth weight [2]. Besides, neonatal sepsis is a main reason for morbidity and mortality in premature infants [3]. Therefore, investigating the molecular mechanisms of neonatal sepsis is urgent and important for developing novel therapies.

The key genes and miRNAs involved in the mechanisms of neonatal sepsis have been reported by several studies. For instance, interleukin (IL)-17A is recognized as a effector of IL-18–mediated injury and the outcomes of neonatal sepsis can be improved by disrupting the IL-18/IL-1/IL-17A axis [4]. Previous studies explore pancreatic stone protein (*PSP*) in neonatal sepsis, finding that PSP is a potential biomarker in combination with procalcitonin in EOS [5, 6]. The overexpression of *CD64* is demonstrated to be a highly specific indicator for patients with neonatal sepsis, and *CD64* index can be utilized to determine LOS before infants show signs of infection [7, 8]. M*iR-15a/16* is a miRNA that may have a critical role in the regulation of gene expression at the post-transcriptional level, which is declared to be useful for the diagnosis and prognosis of neonatal sepsis [9]. In spite of the above studies, the genes and miRNAs acting in neonatal sepsis have not been fully revealed.

In 2014, Cernada et al. [10] used genome-wide expression profiles to assess the expression differences between infants with and without neonatal sepsis, finding that the expression profiles have differences in the early days of neonatal period. However, they have not performed comprehensive bioinformatics analysis to reveal the mechanisms of neonatal sepsis. Using the data deposited by Cernada et al. [10], we further conducted differential expression analysis, enrichment analysis, protein-protein interaction (PPI) network analysis and regulatory network analysis to better or to further investigate the structure and expression of some of the genes that seemed to influence the genesis of neonatal sepsis.

## Materials and methods

### Microarray data

The expression profile data of E-MTAB-4785 was downloaded from the ArrayExpress database (http://www.ebi.ac.uk/arrayexpress/), which was sequenced on the platform of the Affymetrix GeneChip Human Gene 1.0 ST Array [HuGene-1_0-st-v1]. E-MTAB-4785 included 17 blood samples from infants with neonatal sepsis (gestational age = 27 ± 2 weeks, birth weight = 1030 ± 231, 11 males and 6 females) and 19 blood samples from infants without neonatal sepsis (gestational age = 28 ± 2 weeks, birth weight = 1130 ± 329, 13 males and 6 females). All very low birth weight (VLBW) infants were selected from the University and Polytechnic Hospital La Fe between April 2011 to September 2012. Sepsis infants were those with clinical signs of sepsis [11] and/or risk factors [12, 13], while nonseptic infants were those without clinical signs of infection. Venous blood was obtained from sepsis infants and nonseptic infants before starting antibiotics, and stored at − 20 °C for following RNA extraction. The expression profile data of E-MTAB-4785 was deposited by Cernada et al. [10]. The study of Cernada et al. got the approval of the local institutional review board, and informed consent of all participants were provided by their parents or representatives.

### Data preprocessing and differentially expressed genes (DEGs) screening

After the raw data were read by the oligo package (http://www.bioconductor.org/packages/release/bioc/html/oligo.html) [14] in R, data preprocessing were performed using the Robust MultiArray Averaging (RMA) method [15], including background correction, quantile normalization and expression calculation. The average value of probes was obtained as the final gene expression value of their corresponding common gene symbol. The Bayesian testing method [16] in limma package (http://www.bioconductor.org/packages/release/bioc/html/limma.html) [17] was utilized to identify the DEGs between the two group samples. The *p*-values were conducted with multiple testing adjustment based on the Benjamini & Hochberg method [18]. The adjusted *p*-value < 0.05 and |log_2_fold-change (FC)| > 0.585 were set as the thresholds.

### Functional and pathway enrichment analysis

The Gene Ontology (GO, http://www.geneontology.org) project describes gene products from biological process (BP), cell component (CC) and molecular function (MF) aspects [19]. The KEGG (Kyoto Encyclopedia of Genes and Genomes, http://www.genome.ad.jp/kegg/kegg2.html) database involves not only genes but also their functional information [20]. Using the DAVID (Database for Annotation, Visualization and Integrated Discovery, version 6.8, http://david.abcc.ncifcrf.gov) online tool [21], GO functional and KEGG pathway enrichment analyses were carried out for the DEGs. The *p*-value < 0.05 was selected as the cut-off criterion.

### PPI network and module analyses

Using the STRING database (version 10.0, http://string-db.org) [22], the PPI analysis was conducted for the DEGs, with the required confidence (combined score) > 0.4 as the threshold. Followed by the PPI network was visualized by the Cytoscape software (http://www.cytoscape.org/) [23]. Based on the CytoNCA plugin [24] in Cytoscape software, degree centrality (DC), betweenness centrality (BC) and closeness centrality (CC) of nodes were analyzed to identify hub nodes [25]. In addition, module analysis was conducted for the network using the MCODE plugin [26] in Cytoscape software.

### Regulatory network construction

Using the iRegulon plugin [27] in Cytoscape software, the transcription factor (TF)-DEG pairs in the PPI network were predicted, with the Normalized Enrichment Score (NES) > 3 as the threshold. Then, TF-DEG regulatory network was visualized using Cytoscape software [23]. The sepsis-associated miRNAs were searched from the miR2Disease (http://www.mir2disease.org/) database [28], and then their targets were downloaded from the miRWalk 2.0 (http://zmf.umm.uni-heidelberg.de/apps/zmf/mirwalk2/genepub.html) database [29]. Through mapping the target genes into the DEGs, the sepsis-associated miRNA-DEG pairs were screened and then miRNA-DEG regulatory network was constructed by Cytoscape software [23].

## Results

### Analysis of DEGs

Through data preprocessing, the expression values of 18,718 genes were acquired. Compared with normal samples, there were a total of 275 DEGs in the sepsis samples, including 180 up-regulated genes and 95 down-regulated genes. Therefore, there were more up-regulated genes in relative to down-regulated genes.

### Functional and pathway enrichment analysis

GO functional and KEGG pathway enrichment analyses separately were conducted for the up-regulated genes, and the top 5 terms in each category were listed in Table 1. The terms enriched for the up-regulated genes included cellular response to lipopolysaccharide (BP, *p* = 3.05E-06), extracellular exosome (CC, *p* = 3.26E-06), histone acetyltransferase binding (MF, *p* = 2.54E-03), and TNF signaling pathway (pathway, *p* = 3.65E-06). Meanwhile, the top 5 terms enriched for the down-regulated genes were listed in Table 2, including regulation of immune response (BP, *p* = 4.11E-15), T cell receptor complex (CC; *p* = 1.22E-10; which involved zeta-chain-associated protein of 70 kDa, *ZAP70*), transmembrane signaling receptor activity (MF, *p* = 4.20E-06), and T cell receptor signaling pathway (pathway; *p* = 4.90E-07; which involved *ZAP70*).

**Table 1:** The functions and pathways enriched for the up-regulated genes

Category	Description	*P*-value	Gene number
BP	GO:0071222~cellular response to lipopolysaccharide	3.05E-06	10
	GO:0051384~response to glucocorticoid	1.75E-05	8
	GO:0006955~immune response	6.59E-05	14
	GO:0045766~positive regulation of angiogenesis	1.31E-04	8
	GO:0045087~innate immune response	3.22E-04	22
CC	GO:0070062~extracellular exosome	3.26E-06	51
	GO:0005615~extracellular space	3.15E-05	29
	GO:0005886~plasma membrane	1.58E-04	59
	GO:0016020~membrane	2.03E-03	32
	GO:0031093~platelet alpha granule lumen	1.08E-02	4
MF	GO:0035035~histone acetyltransferase binding	2.54E-03	4
	GO:0001077~transcriptional activator activity, RNA polymerase II core promoter proximal region sequence-specific binding	9.14E-03	8
	GO:0002020~protease binding	1.40E-02	5
	GO:0016787~hydrolase activity	1.56E-02	5
	GO:0019899~enzyme binding	1.88E-02	9
Pathway	hsa04668:TNF signaling pathway	3.65E-06	10
	hsa05150:Staphylococcus aureus infection	3.23E-04	6
	hsa05142:Chagas disease (American trypanosomiasis)	7.74E-03	6
	hsa04380:Osteoclast differentiation	1.90E-02	6
	hsa04910:Insulin signaling pathway	2.39E-02	6

*BP* biological process, *CC* cell component, *MF* molecular function

**Table 2:** The functions and pathways enriched for the down-regulated genes

Category	Description	*P*-value	Gene number
BP	GO:0050776~regulation of immune response	4.11E-15	15
	GO:0007166~cell surface receptor signaling pathway	2.56E-06	11
	GO:0050852~T cell receptor signaling pathway	8.71E-05	7
	GO:0072678~T cell migration	3.56E-04	3
	GO:0045059~positive thymic T cell selection	8.47E-04	3
CC	GO:0042101~T cell receptor complex	1.22E-10	7
	GO:0005886~plasma membrane	1.40E-04	35
	GO:0042105~alpha-beta T cell receptor complex	2.26E-04	3
	GO:0001772~immunological synapse	5.25E-04	4
	GO:0009897~external side of plasma membrane	4.83E-03	6
MF	GO:0004888~transmembrane signaling receptor activity	4.20E-06	9
	GO:1990405~protein antigen binding	4.46E-04	3
	GO:0030246~carbohydrate binding	1.62E-03	6
	GO:0023024~MHC class I protein complex binding	1.40E-02	2
	GO:0032393~MHC class I receptor activity	2.31E-02	2
Pathway	hsa04660:T cell receptor signaling pathway	4.90E-07	8
	hsa04672:Intestinal immune network for IgA production	7.58E-05	5
	hsa05340:Primary immunodeficiency	5.41E-04	4
	hsa04514:Cell adhesion molecules (CAMs)	5.95E-04	6
	hsa04650:Natural killer cell mediated cytotoxicity	2.80E-03	5

*BP* biological process, *CC* cell component, *MF* molecular function

### PPI network and module analyses

**Fig. 1:**
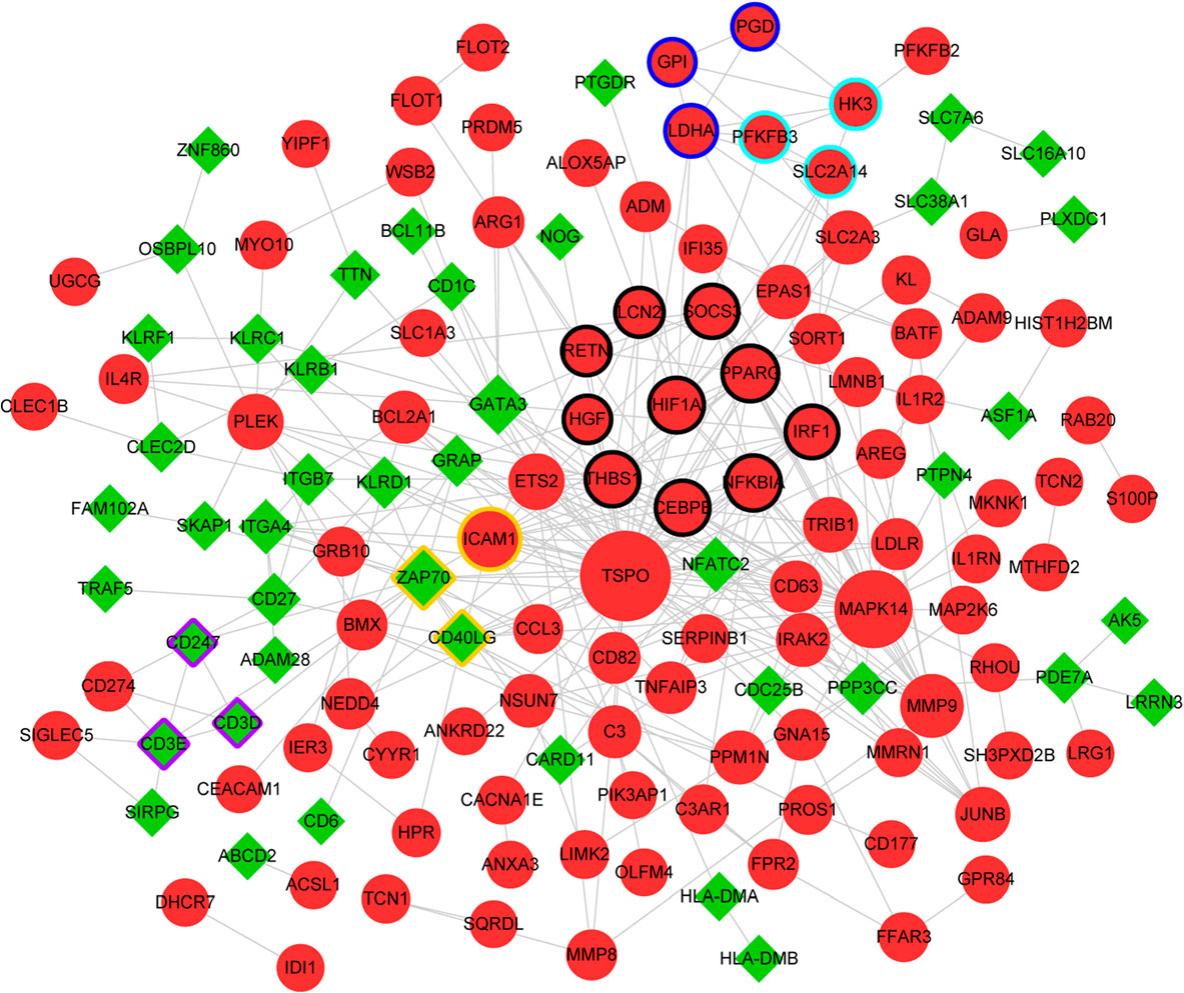
The protein-protein interaction (PPI) network constructed for the differentially expressed genes (DEGs). The red circles and green diamonds separately stand for up-regulated genes and down-regulated genes. The bigger nodes have higher connectivity degrees. Different edge colors represent different modules (module 1: black; module 2: yellow; module 3: dark blue; module 4: purple; module 5: light blue)

A PPI network was visualized for the DEGs, which involved 144 nodes and 311 interactions (Fig. 1). According to the DC, BC and CC scores of nodes, translocator protein (TSPO), mitogen-activated protein kinase 14 (MAPK14), and ZAP70 were the hub nodes in the PPI network (Table 3). Based on the MCODE plugin, a total of 5 modules were identified from the PPI network. Among which, the genes involved in module 1 (which had the highest score, score = 3.333) were mainly enriched in TNF signaling pathway (*p* = 4.59E-03), pathways in cancer (*p* = 5.42E-03), and osteoclast differentiation (*p* = 6.97E-03) (Table 4).

**Table 3:** The top 15 nodes in the protein-protein interaction (PPI) network

Node	DC	Node	BC	Node	CC
TSPO	42	TSPO	8787.401	TSPO	0.084416
MAPK14	30	MAPK14	3945.718	MAPK14	0.082755
ZAP70	18	ZAP70	2945.19	ZAP70	0.081621
ICAM1	18	PLEK	1416.809	ICAM1	0.081481
MMP9	16	CD40LG	1228.869	CD40LG	0.081066
GATA3	13	GATA3	1149.809	NFATC2	0.080882
PPARG	13	PPP3CC	1065.391	THBS1	0.080609
NFKBIA	12	SLC2A3	878.1572	MMP9	0.080609
HIF1A	12	PDE7A	786	NFKBIA	0.080473
CD40LG	11	LDHA	778.0661	GATA3	0.080292
THBS1	11	NFKBIA	706.2174	HIF1A	0.080292
CEBPB	11	GNA15	660.3287	CEBPB	0.080247
SOCS3	10	HIF1A	614.6019	PPARG	0.080157
IRF1	10	CD3E	583.8388	SOCS3	0.080157
PLEK	9	CD82	550.5645	PLEK	0.079844

*DC* degree centrality, *BC* betweenness centrality, *CC* closeness centrality

**Table 4:** The pathways enriched for the genes involved in module 1

Description	Gene number	*P*-value
hsa04668:TNF signaling pathway	3	4.59E-03
hsa05200:Pathways in cancer	4	5.42E-03
hsa04380:Osteoclast differentiation	3	6.97E-03
hsa05160:Hepatitis C	3	7.18E-03
hsa05205:Proteoglycans in cancer	3	1.57E-02
hsa05144:Malaria	2	4.87E-02

### Regulatory network analysis

**Fig. 2:**
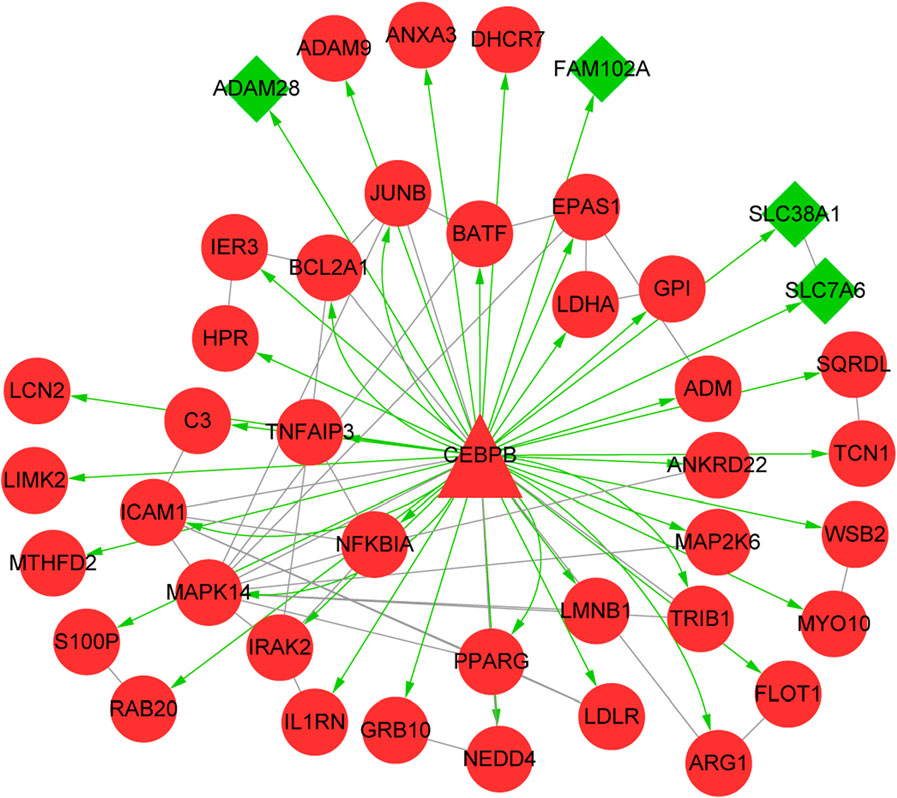
The transcription factor (TF)-differentially expressed gene (DEG) regulatory network identified from the protein-protein interaction (PPI) network. The red circles, green diamonds and red triangles represent up-regulated genes, down-regulated genes and TFs, respectively. Green arrows and grey lines separately indicate TF-DEG regulation relationships and PPIs

A TF-DEG regulatory network was identified from the PPI network, in which the TF CCAAT/enhancer binding protein (C/EBP), beta (*CEBPB*) targeted 43 DEGs (such as *MAPK14*) (Fig. 2). Besides, pathway enrichment analysis was also conducted for the genes involved in the TF-DEG regulatory network, the enriched pathways included TNF signaling pathway (*p* = 4.89E-07, which involved *CEBPB* and *MAPK14*), Epstein-Barr virus infection (*p* = 2.09E-04), and osteoclast differentiation (*p* = 5.64E-04) (Table 5).

**Table 5:** The pathways enriched for the genes involved in the transcriptional regulatory network

Description	Gene number	*P*-value
hsa04668:TNF signaling pathway	7	4.89E-07
hsa05169:Epstein-Barr virus infection	6	2.09E-04
hsa04380:Osteoclast differentiation	5	5.64E-04
hsa04064:NF-kappa B signaling pathway	4	2.19E-03
hsa05145:Toxoplasmosis	4	5.20E-03
hsa04621:NOD-like receptor signaling pathway	3	1.19E-02
hsa05164:Influenza A	4	1.51E-02
hsa05152:Tuberculosis	4	1.58E-02
hsa05140:Leishmaniasis	3	1.94E-02
hsa05142:Chagas disease (American trypanosomiasis)	3	3.93E-02
hsa04620:Toll-like receptor signaling pathway	3	4.07E-02

**Fig. 3:**
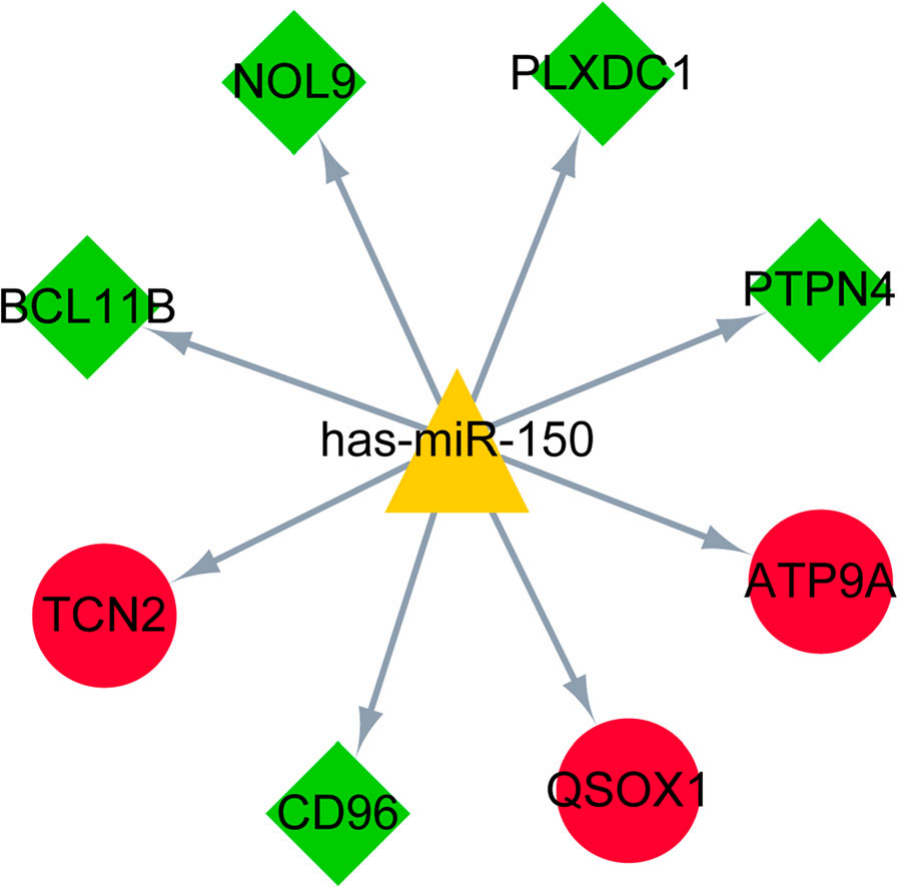
The microRNA (miRNA)-differentially expressed gene (DEG) regulatory network of has-miR-150 and its targets. The red circles, green diamonds and yellow triangles represent up-regulated genes, down-regulated genes and miRNAs, respectively

According to the miR2Disease database, *has-miR-150* was obtained as a sepsis-associated miRNA. Followed by the targets of *has-miR-150* were downloaded from the miRWalk 2.0 database. Among the targets, 8 genes (including 3 up-regulated genes and 5 down-regulated genes; such as B-cell CLL/lymphoma 11B, *BCL11B*) were differentially expressed in this study. In addition, the miRNA-DEG regulatory network is showed in Fig. 3.

## Discussion

In this study, there were 275 DEGs (including 180 up-regulated genes and 95 down-regulated genes) in the sepsis samples compared with normal samples. A PPI network for the DEGs was constructed, in which TSPO, MAPK14, and ZAP70 were hub nodes according to the DC, BC and CC scores. Besides, a total of 5 modules were screened from the PPI network. Furthermore, *CEBPB*-DEG regulatory network and *has-miR-150*-DEG regulatory network separately were constructed.

*TSPO* (also named the peripheral benzodiazepine receptor) functions in the regulation of inflammation and immune function, and its antagonist PK-11195 can relief cigarette smoke extract-induced inflammation [30]. Santoro et al. demonstrate that TSPO ligands can lower pro-inflammatory enzymes and oxidative stress in glial cells via the biosynthesis of neurosteroids, thus these compounds may be used for treating inflammatory-based neuropathologies [31]. The MAPK14/p38α signaling is considered as a central pathway for integrating useful signals in dendritic cells for inflammation and TH17 differentiation [32]. The p38 kinase plays roles in responses of T cells and inflammation, which mediates the production of crucial inflammatory regulators (including *IL-1β*; *TNFα*; and cyclooxygenase-2, *COX-2*) through cells of the innate immune system [33]. The p38 MAP kinase signal transduction pathway plays an essential role in regulating inflammation and proinflammatory cytokine production, and inhibiting *p38α* isoform is sufficient and necessary for anti-inflammatory effect [34]. Enrichment analysis showed that *ZAP70* was enriched in the function of T cell receptor complex and the T cell receptor signaling pathway. The function deletion of the ZAP70 tyrosine kinase in humans can lead to a serious immunodeficiency, characterized by non-functional CD4+ T cells and lacking mature CD8+ T cells [35]. In ZAP70-deficient patients, circulating T cells are under inadequate supervision, no longer differentiate into TH2 T cells, are short of inhibitory growth controls, and show decreased apoptosis, finally developing into inflammation and autoimmunity [36]. These declared that *TSPO*, *MAPK14*, and *ZAP70* might play critical roles in neonatal sepsis.

Through inducing the CEBP family, endoplasmic reticulum (ER) stress results in inflammatory responses in response to palmitic acid stimulation in pancreatic acinar cells, wherein *CEBPB* activation induces *CEBPA* activation [37]. As a key transcription factor affecting metabolic disturbances, *CEBPB* is critical for the maturation and differentiation of adipocytes and is overexpressed during proinflammatory conditions and ER stress [38]. Pathway enrichment analysis indicated that *CEBPB* and *MAPK14* were enriched in TNF signaling pathway. In the TF-DEG regulatory network, *MAPK14* was targeted by *CEBPB*, indicating that *CEBPB* targeting *MAPK14* might function in neonatal sepsis through TNF signaling pathway.

Vasilescu et al. demonstrate that the levels of *miR-150* in both plasma and leukocytes are associated with sepsis aggressiveness and thus can act as a biomarker of early sepsis [39]. Decreased miR-150 serum levels correlate with an adverse outcome in patients with sepsis and critical illness, and circulating miR-150 serum concentrations may be used as a potential prognostic marker in patients with critical illness [40]. Previous studies reported that *BCL11B* is not only a transcription factor specific in T-cell, but also contributes to the lineage fidelity and the genetic and functional programs of mature type 2 innate lymphoid cell (ILC2) [41, 42]. In the miRNA-DEG regulatory network, *BCL11B* was targeted by *has-miR-150*, suggesting that *has-miR-150* might be involved in the pathogenesis of neonatal sepsis by targeting *BCL11B*.

## Conclusions

In conclusion, a total of 275 DEGs were identified from the sepsis samples. Besides, *TSPO*, *ZAP70*, *CEBPB* targeting *MAPK14*, *has-miR-150* targeting *BCL11B* might act in the pathogenesis of neonatal sepsis. However, further experimental researches are needed to confirm these results obtained from bioinformatics analysis.

## Abbreviations

(BP): Biological process
(CC): Cell component
(DC): Degree centrality
(EOS): Early-onset sepsis
(ER): Endoplasmic reticulum
(GO): Gene Ontology
(ILC2): 2 innate lymphoid cell
(LOS): Late-onset sepsis
(MAPK14): Mitogen-activated protein kinase 14
(NES): Normalized Enrichment Score
(PPI): Protein-protein interaction
(PSP): Pancreatic stone protein
(RMA): Robust MultiArray Averaging
(TF): Transcription factor
(TNF-α): Tumor necrosis factor-α
(TSPO): Translocator protein
(VLBW): Very low birth weight
